# Adaptation and validation of the Christian Sanctification of Suffering Scale (CSSS) in a Polish Catholic chronic pain sample

**DOI:** 10.1186/s40359-025-03339-y

**Published:** 2025-08-26

**Authors:** Sebastian Binyamin Skalski-Bednarz, Dariusz Krok

**Affiliations:** 1 Faculty of Philosophy and Education, Katholische Universität Eichstätt-Ingolstadt, Luitpoldstraße 32, 85071 Eichstätt, Germany; 2 Institute of Psychology, Humanitas University, Sosnowiec, Poland; 3 Institute of Psychology, University of Opole, Opole, Poland

**Keywords:** Sanctification of suffering, Spiritual well-being, Chronic pain, Christian psychology, Scale validation

## Abstract

**Background:**

The Christian Sanctification of Suffering Scale (CSSS) measures the extent to which individuals interpret suffering as spiritually meaningful within a Christian framework. This study aimed to adapt and psychometrically validate the CSSS in Polish.

**Methods:**

The study involved a community sample of Polish adults living with chronic pain lasting longer than six months, all identifying as Catholic. Participants completed a 30-minute online survey including the CSSS along with validated measures of religious commitment, spiritual well-being, mental health, and forgiveness. Three months later, a subsample completed the CSSS again to assess temporal stability. The CSSS was translated into Polish and evaluated using confirmatory factor analysis (CFA), item response theory (IRT), reliability analysis, and measurement invariance across sex.

**Results:**

The CSSS demonstrated a unidimensional structure, confirmed by CFA (χ^²^_(27)_ = 39.62, *p* = .056, CFI = 0.941, TLI = 0.921, RMSEA = 0.071, SRMR = 0.033, and GFI = 0.92), and showed excellent internal consistency (α = 0.92). IRT analyses indicated strong item discrimination and a broad range of item difficulty. Measurement invariance across sex was confirmed, and female participants scored significantly higher. Convergent validity was supported through significant correlations with spirituality, religious engagement, mental health indicators, and forgiveness. The scale also demonstrated high temporal stability over a three-month interval.

**Conclusions:**

The Polish version of the CSSS is a psychometrically robust instrument for assessing the sanctification of suffering. It offers a novel instrument for assessing spiritual coping, with potential implications for designing spiritually sensitive clinical interventions.

**Supplementary Information:**

The online version contains supplementary material available at 10.1186/s40359-025-03339-y.

## Background

Sanctification is the process by which individuals perceive certain aspects of life—objects, roles, or experiences—as imbued with sacred significance. According to Pargament and Mahoney [1], *sanctification* may take a theistic form—anchored in a connection with God—or a nontheistic one, involving transcendent or ultimate meanings. Empirical studies have shown that sanctification deepens moral investment, enhances emotional engagement, and intensifies motivational commitment across life domains such as relationships, parenting, sexuality, and embodiment [2–4]. Importantly, sanctification transforms the psychological landscape of whatever is imbued with sacred meaning, reorienting the individual’s cognitive, emotional, and motivational engagement with that domain. In terms of mental processes, this fundamentally alters how people think about, feel toward, and act within the sanctified area of life, often fostering a heightened sense of purpose, commitment, and responsibility. It involves a qualitative shift whereby the sacred becomes not merely an abstract belief but a deeply internalized symbolic framework that elevates personal relevance, emotional significance, and existential depth [1]. Through this lens, ordinary aspects of life−−such as relationships, work, or health−−may take on transcendent importance, serving as conduits for expressing core values, fulfilling spiritual needs, and achieving a sense of connection to something greater than oneself, especially during adversity and illness.

While sanctification has been examined in various domains, its application to suffering is of particular theological and psychological interest. Major world religions conceptualize suffering in divergent ways. In Buddhism, suffering (dukkha) is a universal reality to be transcended through detachment and insight [5]. Hindu traditions emphasize karmic retribution and spiritual liberation through disciplined action [6]. Islamic frameworks generally portray suffering as a divine test (ibtilāʾ) or trial, demanding endurance rather than redemption [7]. Christianity, by contrast, has historically assigned suffering a redemptive dimension—framing it not merely as a burden to be borne, but as a potential site of moral purification, spiritual maturity, and intimate union with the divine [8]. From this perspective, suffering can be regarded as a virtue that predisposes individuals to constructively cope with adversity and overcome pain and discomfort by relying on religious beliefs [9]. It is seen not merely as an unfortunate experience to be avoided but as a meaningful opportunity for personal and spiritual growth as well as getting closer to the divine. In this view, adversity is not wasted but spiritually reinterpreted and integrated into a greater narrative of meaning and transformation.

Such sanctification of suffering reflects more than a coping strategy; it exemplifies a eudaimonic orientation—one that prioritizes spiritual growth, existential meaning, and moral development over mere symptom relief [4, 10]. Within this framework, suffering is not merely endured but spiritually integrated, becoming a catalyst for character formation, deeper relationship with the divine, and narrative coherence. This stands in contrast to hedonic models of well-being, which emphasize distress reduction, and aligns instead with theories of meaning-making, virtue ethics, and self-transcendence [11, 12]. In line with this perspective, Frankl’s logotherapy underscores that discovering meaning and purpose in suffering can transform it into a source of resilience and existential growth [13].

Despite the centrality of suffering in Christian theology and pastoral life, there is a notable lack of validated instruments assessing sacred interpretations of suffering. A structured review by Huang and Wang [14] found only a limited number of psychometric tools appropriate for Christian populations that explicitly measure attitudes toward suffering. This measurement gap not only hinders empirical inquiry but also limits pastoral, clinical, and educational practice. Psychometrically sound measures of religious interpretations of suffering could provide valuable insight into how individuals spiritually integrate hardship and derive purpose, direction, or solace from it.

Among the few instruments that operationalize sacred meaning in suffering, Hall et al. [15] developed the Christian Sanctification of Suffering Scale (CSSS), based on the sanctification framework introduced by Pargament and Mahoney [1]. The CSSS is a unidimensional instrument designed to capture how individuals theologically frame suffering in terms of sacred purpose, divine involvement, and spiritual transformation. The scale demonstrated high internal consistency and a unidimensional factor structure supported through confirmatory factor analysis (CFA). Construct validity was supported through positive associations with religious commitment, intrinsic religiosity, positive religious coping, and spiritual surrender. It also correlated positively with general indicators of well-being, such as flourishing, life satisfaction, and self-efficacy, while showing negative associations with depressive symptoms, anxiety, and negative religious coping. Furthermore, the CSSS aligned closely with sacred themes of suffering such as soul-building, divine providence, and encountering God.

Additional empirical findings underscore the psychological and spiritual implications of sacred appraisals of suffering among Christian populations. Shannonhouse et al. [16] demonstrated that Christian refugees employed theological frameworks emphasizing God’s presence and redemptive purpose, which fostered narrative coherence and emotional resilience in post-traumatic contexts. In a different population, Skalski-Bednarz and Toussaint [17] found that perceiving suffering as sanctified predicted enhanced mental well-being, a relationship partially explained by self-forgiveness. In this context, self-forgiveness functioned as a reconciliatory process—helping individuals integrate difficult experiences into spiritually coherent life narratives, thereby reducing intrapsychic dissonance. Complementing these findings, Kim and Currier [18] showed that perceiving God as obligated to prevent suffering was associated with more intense spiritual struggles, whereas affirming God’s merciful presence in suffering helped mitigate distress. Together, these studies highlight the clinical and existential importance of how individuals interpret divine involvement in adversity.

Although the CSSS offers a robust and theologically grounded measure, it has not yet been adapted for non-English-speaking populations. In Poland, this need is particularly salient given the country’s strong Catholic heritage and the lasting influence of Pope John Paul II—a native Pole—whose teachings have profoundly shaped cultural perceptions of suffering. In Salvifici Doloris [19], he defined suffering as a participation in the mystery of Christ’s Passion that can lead to personal transformation, redemptive purpose, and spiritual solidarity. For many people in Poland, John Paul II’s message framed suffering not as meaningless pain but as a pathway to spiritual maturity, solidarity with others, and participation in the salvific mission of Christ. With over 80% of Poles identifying as Roman Catholic and religious beliefs playing a central role in the interpretation of adversity [20, 21], adapting the CSSS may enhance culturally sensitive assessment and support in both research and clinical contexts.

To assess convergent validity, CSSS scores were hypothesized to correlate positively with religious commitment, spiritual well-being, mental well-being, general health perception, and multiple dimensions of forgiveness—including state forgiveness for others and dispositional forgiveness for self, others, and by God—and negatively with symptoms of depression, anxiety, and stress. These expectations are largely based on the original validation by Hall et al. [15], although the specific relationships between sanctification and these distinct facets of forgiveness remain relatively underexplored (except for associations with self-forgiveness; [17]) and may warrant further theoretical clarification. Conceptually, sanctification frames suffering as spiritually meaningful—where divine mercy meets human limitation and redemptive grace reorients adversity toward growth and reconciliation [1, 22, 23]. Within this framework, forgiveness may be viewed not merely as an emotional resolution but as a spiritually motivated process that fosters self-repair, relational healing, and renewed connection with the divine [24].

## Materials and methods

### Participants

The study involved a community sample of 1,103 Polish adults (67% male; *M*_age_ = 41.7, *SD* = 10.6) living with chronic pain. Data were collected between September and November 2024 in public outpatient clinics specializing in rheumatology, orthopedics, neurology, and pain management, across eight major cities. Participants were recruited through purposive sampling, by scanning a QR code displayed on informational posters in outpatient clinic waiting areas, with recruitment limited to institutions that agreed to display the advertisements. Participation was voluntary, uncompensated, and anonymous. Eligibility criteria included: (a) being at least 18 years old, (b) experiencing chronic pain for a minimum of six months, (c) reporting an average pain level of 4 or higher over the past week on a 0–10 numeric pain scale (0 = “no pain,” 10 = “worst pain imaginable”), (d) fluency in Polish, and (e) identifying as Roman Catholic consistent with the predominant religious affiliation in Poland (see [20]). The survey was administered via the Qualtrics platform. Although no data were collected regarding how many individuals viewed the invitation but did not participate or failed to meet eligibility, all respondents included in the analysis met the eligibility criteria at baseline.

Educational levels were diverse: 15% had primary education, 54% completed vocational or secondary education, and 31% held a university degree. In terms of marital status, 49% were married, 33% divorced, and the remainder were single or widowed. Employment data indicated that 66% of participants were working (full- or part-time), 19% were retired, and 15% were unemployed.

A three-month follow-up was conducted to assess stability over time. A total of 872 individuals (retention rate: 79%) completed the second wave. At follow-up, eligibility was not re-assessed, and all returning participants were included in longitudinal analyses. The study protocol was approved by the University of Economics and Human Sciences in Warsaw (protocol #9/06/22). All participants provided informed electronic consent before participating. Special attention was given to minimizing potential ethical challenges, such as response burden for participants with chronic pain and the sensitivity of questions concerning religious beliefs.

### Procedure

During the first wave of data collection, participants completed a 30-minute online survey. The assessment included demographic and pain-related questions, followed by the CSSS and a series of established self-report instruments measuring religious commitment, spiritual well-being, mental health, and forgiveness. To ensure data quality, response times were recorded and attention checks were embedded in the survey (e.g., “What is 2 + 2?”). No irregularities were detected. At the conclusion of the first wave, participants could optionally provide an email address to be contacted for follow-up. Email addresses were stored separately from survey data to maintain anonymity and confidentiality. A follow-up survey was conducted approximately three months later, in which participants were asked to complete the CSSS only; this took approximately five minutes.

### Measures

#### Sanctification of suffering

Sanctification of suffering was assessed using the Polish adaptation of the CSSS, originally developed by Hall et al. [15]. The CSSS consists of 11 items designed to measure the extent to which individuals ascribe spiritual or divine significance to their experiences of suffering. The scale reflects core Christian beliefs about suffering as a potential avenue for spiritual growth, divine presence, or deepened faith. Example items include “I experience God through my suffering” and “My suffering deepens my relationship with God.” The full list of Polish items is provided in Appendix A. All items are rated on a 7-point Likert scale ranging from 1 (*does not describe at all*) to 7 (*very closely describes*), with higher scores indicating a greater tendency to interpret suffering as sacred or spiritually meaningful. The Polish version of the CSSS was adapted by the authors of the present study with formal permission from the original developers, following a forward–backward translation procedure conducted by a bilingual research team. Semantic accuracy and cultural appropriateness were ensured through expert review by native Polish speakers. Results are reported as mean scores, with higher values reflecting stronger endorsement of religious meaning in suffering.

#### Religious commitment

Religious commitment was measured using the Polish adaptation of the Religious Commitment Inventory–10 (RCI-10 [25, 26]). The RCI-10 comprises 10 items that assess the extent to which individuals integrate religious beliefs and values into their daily lives. It is structured as a single-factor scale, capturing both intrapersonal and interpersonal religious engagement. Responses are provided on a 5-point Likert scale ranging from 1 (*not at all true of me*) to 5 (*totally true of me*). A sample item is: “My religious beliefs underlie my whole approach to life.” The scale demonstrated excellent internal consistency in the present study (α = 0.93).

#### Spiritual well-being

Spiritual well-being was assessed using the Polish version of the Spirituality Index of Well-Being (SIWB [27, 28]). The SIWB includes 12 items that reflect two conceptual areas: self-efficacy and life scheme. In this study, we report the overall global index score. Items are rated on a 5-point scale ranging from 1 (*strongly agree*) to 5 (*strongly disagree*). A sample item is: “I am far from understanding the meaning of life.” The internal consistency of the scale was good (α = 0.83).

#### General health perception

General health was assessed using the EQ Visual Analogue Scale (EQ VAS), which is part of the EQ-5D instrument developed by the EuroQol Group [29]. The official Polish version of the EQ VAS, obtained from the EuroQol Research Foundation, was used in accordance with their guidelines. Participants rated their current overall health on a vertical visual analogue scale ranging from 0 (*the worst health you can imagine*) to 100 (*the best health you can imagine*), in response to the prompt: “Please indicate how good or bad your health is today.” Higher scores indicate better perceived health status. As this is a single-item measure, internal consistency reliability coefficients are not applicable.

#### Mental well-being

Mental well-being was measured using the Polish version of the WHO-Five Well-Being Index (WHO-5 [30, 31]). This 5-item measure assesses the frequency of positive emotional experiences over the past two weeks. The WHO-5 is conceptually and psychometrically established as a single-factor scale. Items are rated on a 6-point scale from 0 (*at no time*) to 5 (*all of the time*). A sample item is: “I have felt cheerful and in good spirits.” In the current sample, internal consistency was high (α = 0.87).

#### Depression, anxiety, and stress

Negative emotional states were assessed using the Polish version of the Depression, Anxiety, and Stress Scale (DASS-21 [32, 33]). The 21 items are divided into three subscales: depression, anxiety, and stress. Participants responded using a 4-point Likert scale ranging from 0 (*did not apply to me at all*) to 3 (*applied to me very much or most of the time*). A sample item is: “I couldn’t seem to experience any positive feeling at all.” The full scale demonstrated excellent internal consistency in this study (α = 0.89).

#### State forgiveness

State forgiveness toward a specific offender was measured using the Polish version of the Rye Forgiveness Scale (RFS [34, 35]). The RFS consists of 15 items capturing both the reduction of negative affect (e.g., anger, resentment) and the emergence of positive attitudes (e.g., empathy, goodwill) toward someone who has caused harm. Participants were instructed to recall a recent situation in which they had been wronged. Items were rated on a 5-point Likert scale from 1 (*strongly disagree*) to 5 (*strongly agree*). A sample item is: “I have been able to let go of my anger toward the person who wronged me.” In this study, we report the total forgiveness score. Internal consistency was high (α = 0.87).

#### Dispositional forgiveness

Dispositional forgiveness was assessed using the Polish version of the Toussaint Forgiveness Scale (TFS [36, 37]). The scale contains 9 items distributed across three subscales: forgiveness for others (5 items), forgiveness for self (2 items), and forgiveness by God (2 items). Items were rated on a 5-point scale from 1 (*strongly disagree*) to 5 (*strongly agree*), with higher scores indicating greater dispositional forgiveness. A sample item is: “I find it hard to forgive myself for some of the things I have done wrong” (reverse-coded). Internal consistency in this study was acceptable for forgiveness for others (α = 0.76), forgiveness for self (α = 0.70), and high for forgiveness by God (α = 0.89).

### Statistical analyses

All statistical analyses were performed using R (version 4.3.2) and relevant packages from the tidyverse and psychometric suites. Descriptive statistics (means, standard deviations, skewness, and kurtosis) were computed for all main study variables. Distributional assumptions were assessed using Shapiro–Wilk tests and visual inspection of histograms and Q–Q plots. No significant deviations were found, supporting the use of parametric procedures. To evaluate the psychometric properties of the CSSS, we conducted CFA. Model fit was assessed with the χ² statistic, CFI, TLI, RMSEA, and SRMR, using conventional cutoffs (CFI/TLI ≥ 0.90 for acceptable fit; RMSEA/SRMR ≤ 0.08) [38]. Internal consistency was estimated using Cronbach’s alpha and McDonald’s omega, and item-level functioning was assessed with corrected item-total correlations. Test–retest reliability over a three-month interval was examined via Pearson’s *r* and paired-samples *t*-tests. Measurement invariance across sex was tested using multigroup CFA at configural, metric, and scalar levels. Additionally, an item response theory (IRT) analysis using the Graded Response Model (GRM) was conducted to examine item discrimination and threshold functioning. Convergent validity was evaluated through Pearson correlations between CSSS scores and related constructs. Although no formal linguistic equivalence analysis was conducted—since this would require a bilingual sample with fluent English skills, which is uncommon in the general Polish population—validity was established through rigorous translation procedures and psychometric evaluation within the current sample. No missing data were present due to forced-response settings. All tests were two-tailed with a significance level of *p* < .05.

## Results

Item-level performance was investigated through corrected item-total correlations, which served to assess the internal consistency of the CSSS items. These correlations were strong, ranging from 0.73 to 0.90, indicating that each item contributed meaningfully to the total scale score. These values, along with the means and standard deviations for each item, are reported in Table 1.

**Table 1:** Item-Level descriptive statistics and CFA parameters for the Christian Sanctification of Suffering Scale (*N* = 1,103)

Item	M (SD)	Item-Total *r*	Loading	SE	z	Standardized Loading
Item 1	2.05 (1.59)	0.73^***^	1.17	0.04	28.29^***^	0.73
Item 2	2.61 (2.03)	0.86^***^	1.77	0.05	36.57^***^	0.87
Item 3	2.20 (1.74)	0.82^***^	1.44	0.04	33.60^***^	0.83
Item 4	1.97 (1.57)	0.88^***^	1.41	0.04	38.43^***^	0.90
Item 5	2.17 (1.77)	0.90^***^	1.64	0.04	40.35^***^	0.92
Item 6	2.27 (1.87)	0.88^***^	1.69	0.04	38.76^***^	0.90
Item 7	2.30 (1.83)	0.90^***^	1.68	0.04	39.99^***^	0.92
Item 8	2.02 (1.62)	0.90^***^	1.48	0.04	39.51^***^	0.91
Item 9	2.06 (1.71)	0.84^***^	1.45	0.04	35.04^***^	0.85
Item 10	1.91 (1.55)	0.84^***^	1.32	0.04	34.99^***^	0.85
Item 11	1.93 (1.60)	0.85^***^	1.38	0.04	35.88^***^	0.86

^***^p < .001

**Fig. 1:**
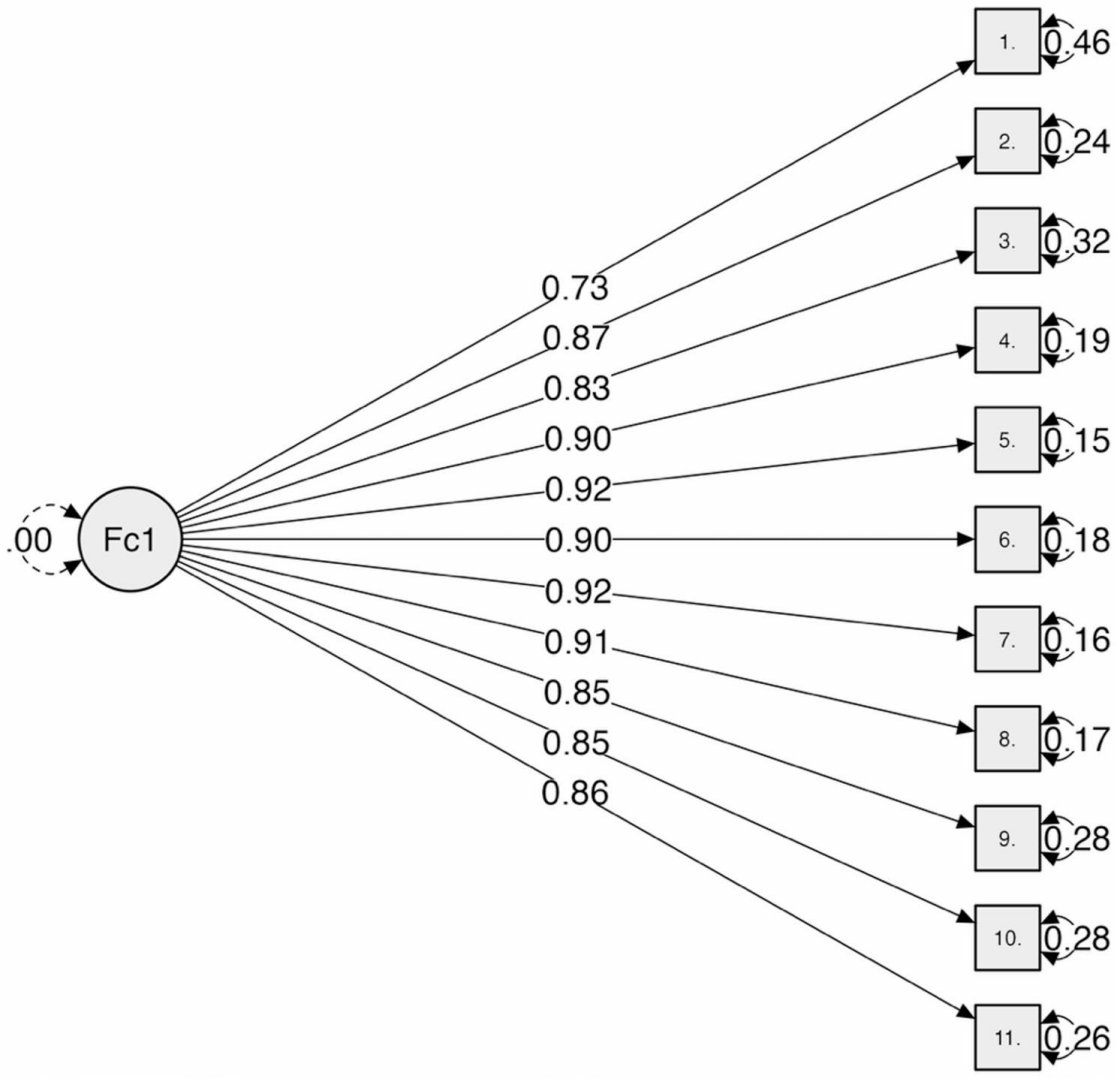
Unidimensional factor structure of the Christian Sanctification of Suffering Scale (*N* = 1,103, all factor loadings are statistically significant at *p* < .001)

Prior to conducting CFA, data suitability was assessed. The Kaiser–Meyer–Olkin (KMO) measure of sampling adequacy was 0.951, indicating excellent shared variance among items. Bartlett’s test of sphericity was significant (χ²_(55)_ = 15369.04, *p* < .001), confirming that the correlation matrix was appropriate for factor analysis. A CFA was conducted using maximum likelihood estimation to test the one-factor structure of the CSSS. The model showed acceptable fit to the data: χ²_(27)_ = 39.62, *p* = .056, CFI = 0.941, TLI = 0.921, RMSEA = 0.071 (90% CI [0.050, 0.089]), SRMR = 0.033, and GFI = 0.92. No model modifications were required, indicating that the one-factor solution adequately accounted for the observed data. Standardized factor loadings ranged from 0.73 to 0.92, indicating strong associations between each item and the latent construct, with all loadings exceeding conventional thresholds for practical significance. The final model structure is depicted in Fig. 1.

Item-level squared multiple correlations (*R*^2^) ranged from 0.54 to 0.85, indicating that a substantial proportion of each item’s variance was accounted for by the latent construct. This range suggests strong item-factor relationships without excessive redundancy.

Measurement invariance was evaluated across sex using configural, metric, and scalar models. The configural model confirmed that the same factor structure held for both groups. Metric invariance (*z* = 1.25, *p* = .211) and scalar invariance (*z* = 0.97, *p* = .331) were both supported, demonstrating that item loadings and intercepts were equivalent across sexes. These findings allow for valid mean comparisons between men and women. Although we relied on chi-square difference testing, it is worth noting that commonly used criteria (ΔCFI ≤ 0.01, ΔRMSEA ≤ 0.015) would also have indicated invariance, given the negligible differences in model fit.

Internal consistency reliability was high. Cronbach’s alpha was α = 0.971 (95% CI [0.968, 0.973]), and McDonald’s omega was ω = 0.970 (95% CI [0.969, 0.974]). Both coefficients indicate strong reliability.

To further examine the psychometric quality of the CSSS items, an IRT analysis was conducted using the GRM [39], which is well-suited for ordinal Likert-type data. This approach allowed for a more refined assessment of item characteristics beyond classical test theory, particularly the items’ precision across the latent trait continuum. Discrimination parameters (α) ranged from 3.19 to 7.69, indicating that all items were highly effective in differentiating individuals with varying levels of sanctification beliefs. The highest discrimination was observed for Items 5 through 8 (α > 7), suggesting strong sensitivity to differences in the underlying construct. Threshold parameters (β₁–β₆) for each item were well-ordered and progressively increasing, indicating appropriate functioning of the 7-point response format and confirming that respondents utilized the categories in a consistent and interpretable manner. Model fit was satisfactory. The model converged successfully, and no local dependence or disordered thresholds were observed. Factor loadings from IRT were in line with the CFA results, with average item loadings above 0.90 (summary: λ = 0.91; *h*^2^ = 0.91), providing further evidence for the unidimensionality and internal coherence of the scale. A full table of item-level parameters from the GRM is presented in Appendix B (Table B1). Overall, the thresholds spanned a wide range of the latent trait, confirming that the CSSS captures variation from low to high levels of sanctification.

Convergent validity of the CSSS was supported by strong positive correlations with forgiveness by God, religious commitment, and spiritual well-being. A moderate positive correlation was observed with dispositional forgiveness for self. Small positive correlations were found with state and dispositional forgiveness for others, as well as mental well-being. A very weak positive correlation was found with general health. Negative correlations were observed with depression (small), stress (small), and anxiety (very weak). All associations were statistically significant, and full coefficients are reported in Table 2.

**Table 2:** Convergent validity of the Christian Sanctification of Suffering Scale (CSSS) (*N* = 1,103)

Variable	M (SD)	Skew	Kurtosis	*r* with CSSS
Religious Commitment (RCI-10)	21.33 (9.76)	0.60	–0.84	0.69^***^
Spiritual Well-Being (SIWB)	32.21 (8.52)	0.35	–0.94	0.52^***^
General Health (EQVAS)	59.21 (14.19)	–0.34	0.53	0.14^***^
Mental Well-Being (WHO-5)	7.86 (5.18)	0.12	–0.20	0.24^***^
Depression (DASS-21)	19.56 (12.50)	0.50	–0.88	–0.23^***^
Anxiety (DASS-21)	13.63 (11.41)	0.68	–0.49	–0.12^***^
Stress (DASS-21)	24.60 (11.46)	0.21	–0.91	–0.18^***^
State Forgiveness				
For Others (RFS)	45.65 (10.24)	–0.29	–0.20	0.23^***^
Dispositional Forgiveness				
For Self (TFS)	6.18 (2.41)	-0.06	–0.99	0.31^***^
For Others (TFS)	13.67 (3.46)	–0.33	–0.37	0.23^***^
By God (TFS)	5.13 (2.84)	0.31	–0.96	0.73^***^
Sanctification of Suffering	23.49 (16.68)	0.93	0.47	--
Level of Pain Severity	5.19 (1.92)	0.05	–0.11	0.17^***^
Duration of Chronic Pain	9.21 (6.54)	0.29	–0.48	0.16^***^
Sex (0 = female, 1 = male)	--	--	--	–0.14^***^

^***^p < .001

To evaluate temporal stability, the test–retest reliability of the CSSS was examined using data from a subsample of 872 participants who completed the measure twice, with a three-month interval between assessments. The mean score at was 24.05 (*SD* = 17.43), and at Time 2 it was 24.20 (*SD* = 17.30). A paired-samples *t*-test indicated that this difference was not statistically significant, *t*_(871)_ = 0.51, *p* = .614. The test–retest correlation was high, *r* = .88, *p* < .001, indicating strong temporal stability of the scale. The associated effect size was minimal (*d* = 0.017, 95% CI [–0.049, 0.083]), further supporting the robustness of CSSS scores over time. In margin, no significant differences were observed between the full baseline sample and the test–retest subsample in terms of distribution or mean levels of the studied variables (*p*_s_ > 0.05), supporting the representativeness of the retested group.

## Discussion

The present study supports the successful adaptation and psychometric validation of the CSSS for use among Polish adults with chronic pain. Rooted in *Sanctification Theory* [1], the CSSS captures a key spiritual process by which individuals attribute sacred meaning to suffering. According to this framework, any domain of life—including pain and hardship—can be “sanctified,” that is, imbued with divine significance and moral weight. This resonates with Christian theological interpretations of suffering as a pathway to spiritual purification, growth, and communion with God, in which suffering is viewed not as meaningless but as a potential source of spiritual depth and grace [8]. The CSSS provides a novel empirical tool to explore the nuanced spiritual meanings people ascribe to suffering [15]. To our knowledge, this is the first validation of the CSSS outside the American context, and our findings suggest it may be applicable in other Christian cultural settings such as Poland. The scale showed excellent internal consistency, construct validity, and test–retest reliability.

CFA and IRT supported the unidimensional structure of the CSSS, consistent with the original validation. Items demonstrated high factor loadings and discrimination parameters, with a broad difficulty range in IRT, indicating precise measurement across varying levels of the sanctification construct.

In terms of convergent validity, the CSSS correlated significantly with religious involvement, spiritual well-being, health indicators, and forgiveness—as both a state and a trait—consistent with literature emphasizing the psychological benefits of sanctifying suffering. Correlations with spiritual well-being were notably stronger than those with self-rated physical and psychological health, suggesting a closer link between sanctification and spiritual identity or existential meaning than with general mental wellness. Importantly, the small-to-moderate effect sizes for correlations with health indicators converge with those reported in the original validation [15], reinforcing the robustness of these findings across cultural contexts. This pattern suggests that sanctification—as a meaning-centered, eudaimonic orientation—may be more strongly linked to preventive and life-affirming outcomes than to symptom reduction [40, 41]. Consistent with this, Hall et al. [15] proposed that sanctification exerts its protective effects via spiritual resilience, enabling individuals to reinterpret suffering in ways that restore coherence and purpose. The strong correlation between CSSS and spiritual well-being in our study supports this framework. Prior research has similarly identified spiritual well-being as a mediator between religious resources and psychological outcomes [42, 43]. These findings also point to possible applications in narrative therapy, helping patients reframe suffering in ways congruent with their religious beliefs and thereby fostering acceptance and facilitating the path toward well-being.

However, interpreting suffering solely as spiritually valuable may pose risks. It could lead to maladaptive patterns, such as glorifying pain, neglecting effective coping strategies, or seeking suffering for perceived spiritual gain [44]. In such cases, sanctification might lose its protective role and contribute to psychological distress, especially in individuals with religious perfectionism or guilt proneness. These individuals may internalize suffering as a necessary condition for worthiness or divine favor, which can lead to fostering chronic self-criticism, shame, and feelings of inadequacy. Moreover, an overreliance on the spiritual merits of suffering may discourage individuals from seeking psychological or medical help, reinforce passive acceptance of harmful circumstances, or heighten vulnerability to depressive symptoms [45]. Therefore, the theme of spiritual suffering should also include a clear psychological understanding that provides a scope for empirical verification. These dynamics warrant further exploration, particularly in clinical populations.

The present results also expand on the findings of Skalski-Bednarz and Toussaint [17], who documented a link between sanctification and self-forgiveness. In this study, significant correlations were also observed with forgiveness for others and perceived forgiveness by God. These findings suggest that a spiritual interpretation of suffering may promote reconciliation not only with the self but also with others and the divine. Morrison’s [22] concept of the “merciful conscience” complements this interpretation, describing conscience as a theophanic encounter with divine grace. This framework—emphasizing openness to mercy and moral transformation—appears particularly resonant with individuals who sanctify suffering, as both constructs underscore the spiritual significance of adversity and the redemptive possibilities it holds. In this light, spiritual tension may be creatively transformed through participation in God’s mercy, thereby enhancing the capacity for forgiveness and deepening relational and spiritual healing.

Measurement invariance confirmed that the CSSS functions equivalently across sex, supporting valid comparisons. Female sex was positively associated with CSSS scores, suggesting that women are more inclined to sanctify suffering and rely on religious coping. This aligns with prior research showing that women engage more in prayer and report stronger religious affiliation than men, particularly within Christian contexts [11, 46]. Recent findings among Polish Catholics also indicate that women, more than men, tend to link their involvement in religious communities with the use of religious coping strategies [47]. Combined with their higher susceptibility to certain chronic conditions and poorer mental health outcomes in the context of pain [48, 49], this may partly account for their stronger inclination to sanctify suffering.

Finally, it should be noted that small but significant correlations were also observed with both pain severity and pain duration, indicating that individuals experiencing more intense or prolonged suffering may be somewhat more likely to ascribe sacred meaning to their condition. This aligns with broader literature on meaning-making and posttraumatic growth, which suggests that framing adversity in spiritual or existential terms often requires time, reflection, and reappraisal to transform distress into perceived growth or purpose [50, 51]. At the same time, the stimulus must reach a certain threshold of intensity to be perceived as threatening and to mobilize coping efforts, making severe or long-lasting pain a context in which sanctification processes may be especially salient [52].

### Limitations and strengths

Some limitations must be acknowledged. The sample was religiously homogeneous, comprising only Roman Catholics, which restricts generalizability to other faith groups. However, this reflects the religious composition of Poland [20]. The cross-sectional design limits causal inferences regarding relationships between sanctification and other variables. Although temporal stability was established, it remains to be examined whether the CSSS is sensitive to changes following spiritual or clinical interventions. Moreover, the nature of suffering—e.g., physical vs. existential—may moderate associations with health outcomes. Finally, discriminant validity was not assessed in the present study, which limits conclusions regarding the distinctiveness of the CSSS from unrelated constructs such as personality traits or social desirability. Taken together, these considerations point to important directions for future research. To overcome these limitations, future studies should recruit more religiously diverse samples, employ longitudinal or experimental designs, and examine the scale across different types of suffering to clarify causal mechanisms and extend generalizability.

Conversely, the study also demonstrates several notable strengths, including the use of a large community–clinical sample, the first validation of the CSSS outside the American context, and the application of advanced psychometric methods. The brevity of the instrument further enhances its feasibility for both research and Catholic pastoral practice.

### Practical implications

For practitioners, the CSSS is a brief and reliable tool that can be applied in clinical settings to assess sacred interpretations of suffering. It bridges psychological science and Catholic pastoral practice, supporting spiritually integrated interventions that recognize both the risks and potential of sanctifying suffering. Validity evidence further suggests that, in pastoral counseling, encouraging this perspective may promote mental health by helping individuals see Christ in their suffering, reinterpret adversity in a redemptive way, and strengthen forgiveness and resilience.

## Conclusions

The present study supports the validity and reliability of the Polish version of the CSSS, a tool measuring the sanctification of suffering in alignment with Christian theological and psychological frameworks. The scale demonstrated a stable unidimensional structure, sound psychometric properties, and measurement invariance across sex. Strong associations with spiritual well-being, religious involvement, and perceived forgiveness by God underscore its relevance to the spiritual-existential domain of coping. While correlations with mental and physical health and other forgiveness dimensions were weaker, the CSSS provides valuable insight into meaning-making processes in chronic pain. These findings highlight its potential for research and spiritually integrated interventions, though further studies should assess its responsiveness to change and generalizability across diverse contexts.

## Electronic supplementary material

Below is the link to the electronic supplementary material.

Supplementary Material 1

Supplementary Material 2

## Data Availability

The data presented in this study are available from the corresponding author upon reasonable request for academic and non-commercial research purposes. Requests must include a clear research plan, and access will be granted in accordance with privacy considerations and subject to approval by the institutional ethics committee overseeing the study.

## Abbreviations

CFA: Confirmatory Factor Analysis
CI: Confidence Interval
CSSS: Christian Sanctification of Suffering Scale
DASS-21: Depression, Anxiety, and Stress Scale – 21 Items
EQ VAS: EQ Visual Analogue Scale
GFI: Goodness-of-Fit Index
GRM: Graded Response Model
IRT: Item Response Theory
KMO: Kaiser–Meyer–Olkin Measure
RCI-10: Religious Commitment Inventory–10
RFS: Rye Forgiveness Scale
SD: Standard Deviation
SIWB: Spirituality Index of Well-Being
TFS: Toussaint Forgiveness Scale
WHO-5: WHO-Five Well-Being Index
