# Supplementary material for: Adaptation and validation of the Christian Sanctification of Suffering Scale (CSSS) in a Polish Catholic chronic pain sample
Source: BMC Psychol. 2025 Aug 26;13:966. doi: 10.1186/s40359-025-03339-y (PMC12379455; doi:10.1186/s40359-025-03339-y)
Supplement: Supplementary file 1 — Supplementary Material 1 [file 40359_2025_3339_MOESM1_ESM.docx]

**Appendix A**

*Polish Statement Items of the Christian Sanctification of Suffering Scale (CSSS)*

1. Moje cierpienie jest odzwierciedleniem woli Boga. [My suffering is a reflection of God’s will]

2. Bóg jest obecny w moim cierpieniu. [God is present in my suffering]

3. Bóg wykorzystuje moje cierpienie do realizacji swoich celów. [God uses my suffering to accomplish God’s purposes]

4. Przez moje cierpienie mogę wielbić Boga. [God is glorified through my suffering]

5. Doświadczam Boga poprzez moje cierpienie. [I experience God through my suffering]

6. Cierpienie nabiera sensu, gdy powierzam je Bogu. [Expressing my suffering to God helps redeem my suffering]

7. Moje cierpienie pogłębia moją relację z Bogiem. [My suffering deepens my relationship with God]

8. Bóg przemawia do mnie poprzez moje cierpienie. [God speaks to me through my suffering]

9. Bóg przemawia do mnie przez Biblię w moim cierpieniu. [God uses the Bible to communicate to me in my suffering]

10. Moje cierpienie czyni mnie bardziej podobnym do Chrystusa. [My suffering makes me more Christlike]

11. Moje cierpienie jednoczy mnie z cierpieniem Jezusa Chrystusa. [My suffering unites me with the sufferings of Jesus Christ]
