# Supplementary material for: Adaptation and validation of the Christian Sanctification of Suffering Scale (CSSS) in a Polish Catholic chronic pain sample
Source: BMC Psychol. 2025 Aug 26;13:966. doi: 10.1186/s40359-025-03339-y (PMC12379455; doi:10.1186/s40359-025-03339-y)
Supplement: Supplementary file 2 — Supplementary Material 2 [file 40359_2025_3339_MOESM2_ESM.docx]

**Appendix B**

**Table B1**

*Item Parameters from the Graded Response Model for the Christian Sanctification of Suffering Scale (CSSS; N = 1103)*

| **Item** | **Discrimination (α)** | **β₁** | **β₂** | **β₃** | **β₄** | **β₅** | **β₆** |
| --- | --- | --- | --- | --- | --- | --- | --- |
| Item 1 | 3.19 | 0.36 | 0.72 | 1.01 | 1.35 | 1.70 | 2.15 |
| Item 2 | 4.92 | 0.10 | 0.44 | 0.66 | 0.94 | 1.19 | 1.45 |
| Item 3 | 4.33 | 0.29 | 0.63 | 0.89 | 1.17 | 1.47 | 1.76 |
| Item 4 | 6.95 | 0.47 | 0.74 | 0.96 | 1.27 | 1.51 | 1.76 |
| Item 5 | 7.69 | 0.40 | 0.66 | 0.86 | 1.14 | 1.37 | 1.57 |
| Item 6 | 6.49 | 0.32 | 0.66 | 0.86 | 1.09 | 1.29 | 1.50 |
| Item 7 | 6.68 | 0.29 | 0.60 | 0.82 | 1.08 | 1.33 | 1.57 |
| Item 8 | 7.60 | 0.44 | 0.73 | 0.92 | 1.23 | 1.47 | 1.69 |
| Item 9 | 4.99 | 0.44 | 0.76 | 0.95 | 1.25 | 1.44 | 1.67 |
| Item 10 | 5.18 | 0.53 | 0.81 | 1.01 | 1.30 | 1.60 | 1.87 |
| Item 11 | 5.72 | 0.55 | 0.80 | 0.98 | 1.30 | 1.51 | 1.77 |
